# The effects of water temperature on physiological responses and exercise performance during immersed incremental exercise

**DOI:** 10.1186/2046-7648-4-S1-A37

**Published:** 2015-09-14

**Authors:** Tomomi Fujimoto, Yosuke Sasaki, Hitoshi Wakabayashi, Yasuo Sengoku, Shozo Tsubakimoto, Takeshi Nishiyasu

**Affiliations:** 1 Graduate School of Comprehensive Human Sciences, University of Tsukuba, Japan; 2 Faculty of Engineering, Chiba Institute of Technology, Japan; 3 Institute of Health and Sport Sciences, University of Tsukuba, Japan

## Introduction

Aquatic exercise such as swimming is performed in the water of 18 to 34 °C because of the differences in ambient environmental conditions. Heat conductivity of water is greater than that of air, therefore water temperature would have a considerable impact on physiological responses and exercise performance. A previous study has shown that, oxygen consumption (V(·)O_2_) during immersed cycle exercise at submaximal workload is greater in cold water (18 °C) compared to moderately cool and warm water (25 and 34 °C) [1]. Furthermore, previous studies have reported decreased V(·)O_2 _at maximal work (V(·)O_2peak_) in the cold water [2], while others have reported no change [3]. Therefore, consensus views on whether difference in water temperature affects V(·)O_2peak _and exercise performance hasn't been obtained. The purpose of this study was to investigate the effects of water temperature on physiological responses and exercise performance using immersed incremental cycle exercise until exhaustion.

## Methods

Ten healthy young men performed incremental exercise on a water cycle ergometer in a semi-recumbent position. The subjects immersed to their shoulders and performed the exercise in 3 water temperatures (Tw): 18, 26 and 34 °C. For the exercise, initial workload was 60W and increased 20 W every 2 minutes at first 4 levels, and then increased 10 W every minute until they would no longer continue. The workload was increased by electrical brake whilst keeping a constant pedalling rate (60 rpm) in an attempt to avoid changes in the water external force exerted on the legs. Oesophageal temperature, skin temperature, expired gases, heart rate and maximal workload were measured. This research conformed to the principles of the Declaration of Helsinki, and all subjects signed an informed consent form.

## Results

During submaximal exercise (60 to 120 W), V(·)O_2 _was higher in T_w _18 compared to other conditions (T_w _26 and 34). While maximal workload in T_w _18 was lower than in the other conditions (T_w _18 mean (SD): 138(16) W, T_w _26: 157(15) W, T_w _34: 156(18) W), V(·)O_2peak _did not differ among conditions (T_w _18: 3156(364) mL.min^-1^, T_w _26: 3270(344) mL.min^-1^, T_w _34: 3281(268) mL.min^-1^). Minute ventilation during maximal and submaximal exercise and tidal volume during submaximal exercise were higher in T_w _18 compared to the other conditions, while respiratory frequency did not differ between conditions.

## Discussion

The lower maximal workload in T_w _18 may be due to the fact that, even though V(·)O_2peak _was same level among all conditions, it reached peak values faster in T_w _18 compared to the other conditions, since V(·)O_2 _in T_w _18 during submaximal exercise was already elevated. The enhanced ventilatory response in T_w _18 was the result of the enhanced tidal volume rather than respiratory frequency.

## Conclusion

During immersed incremental cycle exercise, exercise performance decreases in cold water (18 °C ) due to V(·)O_2 _reaching peak values faster. Ventilatory response (Vt) is enhanced in cold water (18 °C).
